# Using bioluminescence to image gene expression and spontaneous behavior in freely moving mice

**DOI:** 10.1371/journal.pone.0279875

**Published:** 2023-01-20

**Authors:** Astha Malik, Jessica A. Zavadil, Michael E. Geusz

**Affiliations:** 1 Division of Gastroenterology, Hepatology, & Nutrition, Cincinnati Children’s Hospital Medical Center, Cincinnati, Ohio, United States of America; 2 Graduate Medical Education, University of Tennessee Health Science Center, Memphis, TN, United States of America; 3 Department of Biological Sciences, Bowling Green State University, Bowling Green, Ohio, United States of America; Karlsruhe Institute of Technology, GERMANY

## Abstract

Bioluminescence imaging (BLI) of gene expression in live animals is a powerful method for monitoring development, tumor growth, infections, healing, and other progressive, long-term biological processes. BLI remains an effective approach for reducing the number of animals needed to monitor dynamic changes in gene activity because images can be captured repeatedly from the same animals. When examining these ongoing changes, it is sometimes necessary to remove rhythmic effects on the bioluminescence signal caused by the circadian clock’s daily modulation of gene expression. Furthermore, BLI using freely moving animals remains limited because the standard procedures can alter normal behaviors. Another obstacle with conventional BLI of animals is that luciferin, the firefly luciferase substrate, is usually injected into mice that are then imaged while anesthetized. Unfortunately, the luciferase signal declines rapidly during imaging as luciferin is cleared from the body. Alternatively, mice are imaged after they are surgically implanted with a pump or connected to a tether to deliver luciferin, but stressors such as this surgery and anesthesia can alter physiology, behavior, and the actual gene expression being imaged. Consequently, we developed a strategy that minimizes animal exposure to stressors before and during sustained BLI of freely moving unanesthetized mice. This technique was effective when monitoring expression of the Per1 gene that serves in the circadian clock timing mechanism and was previously shown to produce circadian bioluminescence rhythms in live mice. We used hairless albino mice expressing luciferase that were allowed to drink luciferin and engage in normal behaviors during imaging with cooled electron-multiplying-CCD cameras. Computer-aided image selection was developed to measure signal intensity of individual mice each time they were in the same posture, thereby providing comparable measurements over long intervals. This imaging procedure, performed primarily during the animal’s night, is compatible with entrainment of the mouse circadian timing system to the light cycle while allowing sampling at multi-day intervals to monitor long-term changes. When the circadian expression of a gene is known, this approach provides an effective alternative to imaging immobile anesthetized animals and can removing noise caused by circadian oscillations and body movements that can degrade data collected during long-term imaging studies.

## Introduction

Bioluminescence imaging (BLI) methods based on firefly luciferase are remarkably effective for monitoring gene regulation, the location of gene expression within organisms or tissues, its magnitude and duration. When performed with living cells or organisms, BLI provides distinct advantages over fluorescence methods [1, 2]. BLI of live mice expressing luciferase also reduces the number of animals needed for research by allowing the time-course of an ongoing physiological process to be measured through repeated imaging of individual mice [3–6]. In contrast, when similar information is obtained by harvesting tissue, several mice are required at each time point to describe the dynamics of gene expression over several days. Repeated BLI is therefore most valuable for long-term studies when, for example, examining development, circadian rhythms, healing, tumor growth, and metastasis while also providing comparative data from multiple body regions simultaneously. As described previously [7], BLI is considerably less expensive than other whole-animal imaging techniques such as SPECT, PET, and MRI that require more sophisticated equipment.

In typical BLI experiments mice are imaged quickly, with several-minute exposures, so that many mice can be imaged throughout the day. Along with this rapid throughput, mice are then imaged again at roughly one or two-day intervals to observe changes such as the spread of an infection or an inoculation that was engineered to produce bioluminescence [7]. An obvious problem with this approach in respect to the circadian timing system of the animal is that the luciferase signal may vary throughout the day, and this oscillation may add unwanted noise to the signal of interest. Furthermore, the handling and constraint of mice while injecting luciferin, the luciferase substrate, is a stressor that could disrupt the circadian timing system, causing shifts in the phase of its component circadian oscillators within the body. Stress from physical constraint elevates blood corticosterone levels in animals [8] and may alter the gene expression patterns being imaged. Chronic stress has also been shown to increase tumor growth in mice [9] and suppresses both wound healing [10] and immune activity [11]. Other reported stressors to consider include surgical implantation of pumps or optical fibers and windows, transport to and from the animal care facility, the change in environment, time away from the home cage, disturbances from auditory noise particularly at ultrasonic frequencies, and major changes in ambient lighting that can disrupt the phase of the animal’s circadian clock [12]. Therefore, there is an urgent need to develop innovative and novel methods to allow continuous imaging of conscious, unrestrained mice, ideally without surgical interventions in a controlled low stress setting much like that of the animal’s home cage.

During standard BLI, mice are immobilized with anesthesia to minimize most of the blurring caused by body movements [1]. To provide faster imaging, cooled charge-coupled device (CCD) cameras have been replaced with intensified-CCD cameras or cooled electron-multiplying CCD (EMCCD) cameras. Nevertheless, currently available bioluminescence methods are not optimal for sustained measurements of gene expression in freely moving mice as they display normal behaviors. Instead, unconscious mice are imaged in an immobilized state using anesthetics that, unfortunately, interfere with physiology and luciferase enzyme activity. Isoflurane, a commonly used anesthetic for BLI, is reported to suppress light production by as much as half [13]. Some of these effects can be reduced by careful anesthetic dosing and advanced respiration monitoring, although this is not typically performed [14]. In addition, care is warranted when studies use luciferase-based imaging to explore genes and proteins involved in learning and memory because isoflurane inhibits neurogenesis and spatial memory in mice [15]. Loss of normal thermoregulation from anesthesia requires constant regulation of body temperature during imaging, and temperature fluctuations, along with altering metabolism, significantly affect luciferase signal intensity and results [1, 5, 16].

Some reports have described BLI of non-anesthetized, freely moving mice [13, 17–19], but have not fully resolved the question of how best to introduce luciferin (D-luciferin), the non-toxic water-soluble luciferase substrate. Commonly used procedures, intraperitoneal (IP) or tail vein luciferin injection, alter ongoing mouse behavior [20], require restraint during handling, and more importantly provide only a transient window of approximately 10–15 min of optimal imaging. Nevertheless, when luciferase signals are always imaged at a specific time after injection, these gene expression assays are repeatable and accurate [2]. Interestingly, the slow luciferase reaction rate causes only small amounts of luciferin to be consumed during long time intervals, as shown by experiments involving luminometry of cell cultures, organoids or tissue explants that are performed without the need to replenish luciferin for several days [6, 21–23]. Furthermore, BLI is advantageous because the low flux of emitted photons from the luciferase reaction is unlikely to influence cellular processes or viability, unlike the risk from excessive excitation light during fluorescence-based assays [24].

Freely moving BLI allows measurements of gradual changes in gene expression and efficient monitoring of disease processes and circadian cycles. Circadian clocks found throughout animals generate daily rhythms in cell division, healing, metabolism, and physiological processes that can also impact the progression of cancer and various disorders [25–32]. Depending on the cell type, circadian clocks directly control around 10–40% of gene activity [33] and are particularly relevant to BLI studies. Circadian clocks depend on daily transcription of core clock genes such as Per1 and Per2 in mice and their orthologs in other species [34, 36].

Circadian rhythms in Per2 expression have been recorded in freely moving hairless albino hr-/hr- mice (SKH1) using an EMCCD camera and a photomultiplier tube that collected an integrated luciferase signal while luciferin was supplied through an implanted pump [17]. A recent study described circadian rhythms in expression of the D-site albumin promoter binding protein gene measured in freely moving Cre-recombinase-dependent mice by BLI after oral luciferin delivery [35]. Another research group measured circadian rhythms in gene expression by repeatedly anesthetizing and imaging mice during a single day, although they were unable to correct for any stress effects on gene expression and the circadian clock in response to frequent handling and anesthesia [36]. Of particular concern, studies have shown how physiological stressors can shift the phase of the circadian corticosteroid rhythm that controls daily rhythms in gene expression in the body [37–39]. Other external stimuli include daily cycles of light, sound or social activity that also shift the phase of circadian clocks by acting as Zeitgebers (time givers) to entrain the circadian clock system [39–41].

A sophisticated method has been developed to precisely measure circadian rhythms in expression of the core clock gene Per1 in areas of freely moving mice [42]. Images of fluorescent dots attached to dorsal body areas were used to calculate the distance from these point sources to the camera and correct for bioluminescence signal loss with distance and position. A remarkable spatial resolution was reported, less than 2 mm, but the mice remained tethered to a luciferin delivery system and were subjected to surgery before imaging. These physical interventions suggest that completely normal behaviors and the full mobility of mice like that in their home cage may not have been observable. Furthermore, the system is more complicated and expensive than typical imaging systems, requiring two EMCCD cameras and UV illumination to measure specific body locations.

In contrast, we sought to develop a relatively simple and affordable BLI system using existing technologies for truly freely moving mice displaying normal behaviors while completely unrestrained within typical housing conditions. An additional goal was to find ways to reduce the influence of circadian rhythms on the bioluminescence signal by considering it a source of noise to be eliminated in studies addressing processes occurring over longer time intervals such as development, tumor growth, or infections and their associated behaviors. We suggest a protocol that should minimize effects of the BLI procedure on the circadian system and control for effects of the circadian system and body movements on the BLI signal. Essentially, in the optimized procedure mice are removed from the colony at the end of the day while still in the light and placed into darkness continuing into the following day during which time they are imaged repeatedly and allowed to assimilate luciferin by drinking. Mice are then returned to their home cages near or just after dawn to resume entrainment of their circadian system to the light/dark cycle and for any social interaction with their cage mates.

To test the limits of BLI for monitoring gene expression in live, conscious, unrestrained animals we used hairless albino transgenic mice (Hr-Per1) that contain the firefly gene luc expressed under control of the Per1 gene promoter. The lack of both fur and pigmentation improves signal detection and the signal-to-noise ratio [5, 43]. Hairless mice provide remarkably clear views of the skin and nearby underlying structures such as the submandibular salivary gland (SMG), mammary glands, muscles, tail vertebrae, and pancreas [44]. Because of their fast responsiveness, immediate-early genes such as Per1 are well suited for freely moving BLI. We describe how luciferin delivered through voluntary drinking in combination with computer-aided image selection (CAIS) enables BLI of mice engaged in typical activities free of external stressors [45]. Although hairless mice were used here, we feel this method is also suitable for imaging a wide range of mouse strains, particularly while they perform normal behaviors.

## Materials and methods

### Hairless mice

Hr-Per1 mice were bred and maintained in temperature-controlled rooms, fed ad libitum, and were exposed to standard cycles of 12 hrs light/12 hrs darkness to entrain their circadian system. Male and female adult mice were used for imaging. The Hr-Per1 line was produced through a cross between HRS/J hairless, albino mice (stock number: 000673, The Jackson Laboratory, Bar Harbor, ME) and Per1::luc mice that contain the firefly gene luc expressed under control of the promoter for the core circadian clock gene period 1 (Per1). The Per1::luc mice are on a C57BL/6 background and from a colony at BGSU started from transgenic mice made by Dr. Hajime Tei of the Mitsubishi Kagaku Institute of Life Sciences, Tokyo [46]. After repeated selection and crosses between the progeny, a mouse line was selected that was homozygous for albinism and the hr gene mutation producing permanent hair loss near the time of weaning [47]. To increase breeding efficiency the line was then crossed with the hairless, albino SKH1 mouse line (Crl:SKH1-Hr^hr^, Charles River, Wilmington, MA). SKH1 mice have a competent immune system [48], unlike the hairless, albino “nude” mice (nu/nu) used in many tumor and imaging studies. All procedures were approved by the Bowling Green State University Institutional Animal Care and Use Committee and met guidelines for use of laboratory animals of the National Institutes of Health and the Guide for the Care and Use of Laboratory Animals. All efforts were made to minimize animal discomfort.

### Imaging anesthetized, unconscious mice

Hr-Per1 mice were injected IP with luciferin (Caliper Life Sciences, Hopkinton, MA) dissolved in physiological saline (0.1 ml, 10 mM). Sedation was produced with 3% isoflurane in oxygen in a Plexiglas induction chamber (E-Z anesthesia, Euthanex, Palmer, PA) that was also used during imaging through a removable glass window in the top of the induction chamber. Body temperature was maintained with an isothermal pad (Braintree Scientific, Braintree, MA). Eight mice maintained under anesthesia were imaged. When mice were in the dark phase of their cycle, they were brought to the imaging station in a light-proof box and illumination was provided by red LED lights.

Ten minutes after luciferin injection, mice were positioned on their side for imaging with a liquid nitrogen-cooled CCD camera (CH-360 Teledyne Photometrics, Tucson, AZ) containing a back-thinned, back-illuminated sensor cooled to -91°C to eliminate all dark current and a 50-mm f/1.2 Nikkor lens (Nikon, Melville, NY), as described previously [45]. One-minute exposures and 4 x 4-pixel on-chip binning of the camera sensor were used to improve signal capture and reduce camera read noise. Images were collected and analyzed with V^++^ (Digital Optics) and ImageJ (NIH) software version 1.51j8. Reference images were captured in red LED light. Bioluminescence intensity was presented as analog-to-digital units (ADUs) of the camera sensor.

### Imaging freely moving mice

The method for delivery of luciferin in drinking water was described previously [45] and was combined with a specially designed chamber (S1 Fig). An optical window was placed over the top of a cylindrical black plastic chamber, composed of ABS (acrylonitrile, butadiene, styrene), with a diameter matching the camera field of view (about 12 cm wide) from above. A total of 10 Hr-Per1 mice (4 males, 6 females) were used for freely moving BLI. The camera was in a darkroom with the camera controls located outside, as described above. Foam spacers below the optical window and a lower hole provided air flow. Depending on the imaging session duration, 1 mM luciferin (potassium salt) in 10 ml pure, bottled apple juice was provided in either a 60-mm glass petri dish or a plastic 15-ml centrifuge tube (Corning). The tube was connected through a rubber stopper to a standard metal sipping tube used with mouse water bottles, which extended through the container wall (S2A Fig). A second hole opposite the tube was used for ventilation. The volume consumed was monitored from outside the chamber. Mice typically drink less than 20 ml per day [49].

Imaging lasted up to 24 hours during which the drinking tube was refilled at about 10-hr intervals while in red LED light. Otherwise, mice were in continuous darkness. Ambient temperature was maintained near 25°C. Food pellets (Teklad S-2335) and ground corn cob bedding were also included in the chamber. The space provided by the chamber was more than that recommended for even a large, 50-gram mouse (15 square inches) according to the Guide for the Care and Use of Laboratory Animals (National Institutes of Health, Bethesda, MD, USA) [50].

Freely moving mice were imaged with the cooled-CCD camera (CH360) or two EMCCD cameras that had similar quantum efficiency, pixel well depth, and pixel size. The first EMCCD camera contained a back-illuminated 512 x 512-pixel sensor cooled to -80°C (Evolve, Teledyne Photometrics, Tucson, AZ). EM gain for photon counting mode was set between 20x and 400x with 5 MHZ readout. The on-chip gain was varied to optimize the signal/noise ratio during 50-ms to 5-sec exposures. The second camera (iXon3, Andor Technology, South Windsor, CT) was tested with EM gain between 700x and 1000x, preamplifier gain at 3, and Kinetic mode. On-chip pixel binning between 2 x 2 and 8 x 8 was evaluated. Neither of the EMCCD cameras had a mechanical shutter that would produce noise. Both were equipped with the same 50-mm lens used with the CH360 camera.

### Image processing and analysis

Images were typically captured at one-second intervals in a time series lasting several minutes to hours. The 16-bit camera images were processed by first removing the camera bias from each image in all stacks of images, each stack being a time series. Several time series were collected from each mouse. An average of 20 bias-only image frames (sent from the camera without an exposure) was subtracted from each raw image to remove this non-image background noise. A median filter (3 x 3-pixel kernel or 2-pixel diameter using V++ or ImageJ, respectively) was used to remove noise caused by spurious charge and cosmic ray-related events as described in detail in S1 File, resulting in the initially processed image stacks (time series). Distinct extended or curled postures were first identified by eye in all frames of one initial image stack resulting in two selected image sets. “Extended” was when the mouse was upright with feet on the chamber floor. “Curled” mice were also positioned with their back facing up but with the torso curled downward creating a more rounded object. ImageJ was then used to calculate average roundness values of mice in the two eye-selected sets that were based on posture (S1 File).

Before calculating roundness, the thresholding feature was used to convert the median-filtered 16-bit images into binary images with only black or white pixels, which is needed to identify the object edge and roundness. Integrated Intensity measurements, on the other hand, were calculated using the initially processed images, which are grayscale rather than binary. For images captured using the photon-counting mode of the iXon3 camera, we used an alternative CAIS procedure described in S1 File. Essentially, a Gaussian blur function was applied to images of mice, followed by thresholding to generate a binary image, and then an ellipse was fit using ImageJ to provide roundness measurements.

Average roundness for eye-selected extended and curled mice was then used to create two acceptable roundness ranges for use in the CAIS procedure to automatically categorize mice in images of additional time series into one of the two postures. Mouse images with roundness outside the two ranges were automatically excluded from further analysis, reducing the length of the time series while removing frames that would contribute to the noise because the mice were in different postures. ImageJ roundness data and frame numbers in the two sets were saved and then opened in Excel. The data were sorted by roundness values from low-to-high, and then the corresponding frames falling in the extended and curled ranges were selected along with their roundness and frame numbers. The average roundness in both categories was calculated. The frame numbers for images in both categories were used to create two new time series in ImageJ, using Make Substack, so that average integrated intensity could be calculated from the corresponding images in the original initially processed time series before they were converted to binary images. The signal-to-noise ratio was calculated in Excel as the integrated intensity averaged across all frames in the time series (either original or selected series) divided by the standard deviation of the average integrated intensity.

Located among the ImageJ Shape Descriptors in the Measure function, the roundness parameter varies between 1 (a perfect circle) and 0, and it is the inverse of the aspect ratio of an ellipse fit to the object identified by particle analysis. To minimize error from edge effects, we also tested whether exclusion from the analysis of objects identified as mice that had center-of-mass coordinates outside the central quadrant would improve imaging accuracy. For images with only 256 x 256 pixels, we used a criterion that Center of Mass must have coordinates with both x (XM) and y (YM) greater than 63 and less than 193.

## Results

### Freely moving versus unconscious Hr-Per1 mouse bioluminescence imaging

**Fig 1:**
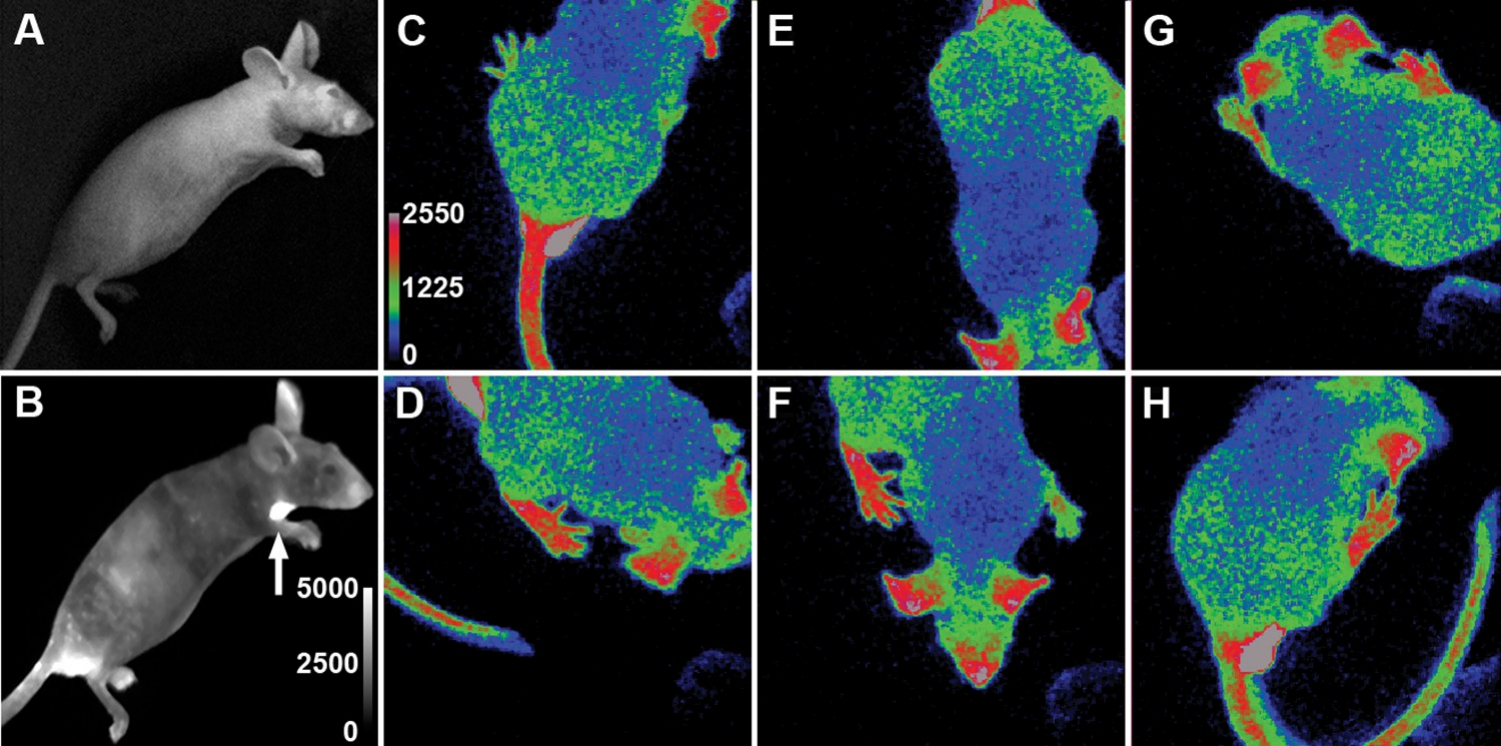
Freely moving mice and unconscious mice imaged after intraperitoneal luciferin injection. **A:** Brightfield image of an immobile (anesthetized) male Hr-Per1 mouse. **B:** Corresponding bioluminescence alone after 10-min image capture of the same mouse injected with luciferin showing high expression in several body areas including the submandibular gland (arrow) and testes. **C-H:** Bioluminescence in a freely moving male mouse after luciferin injection. Frames were selected to show various body postures captured during a 1-min sequence of 0.5 second images collected with 100x EM gain and 2 x 2-pixel binning. The entire body is visible and shows high gene expression in paws, ears, snout, and tail. Signal in the testes (**C**&**H**) is higher than the intensity range shown by the scale. The intensity ranges shown are in analog-to-digital units (ADUs) of the CCD camera.

**Fig 2:**
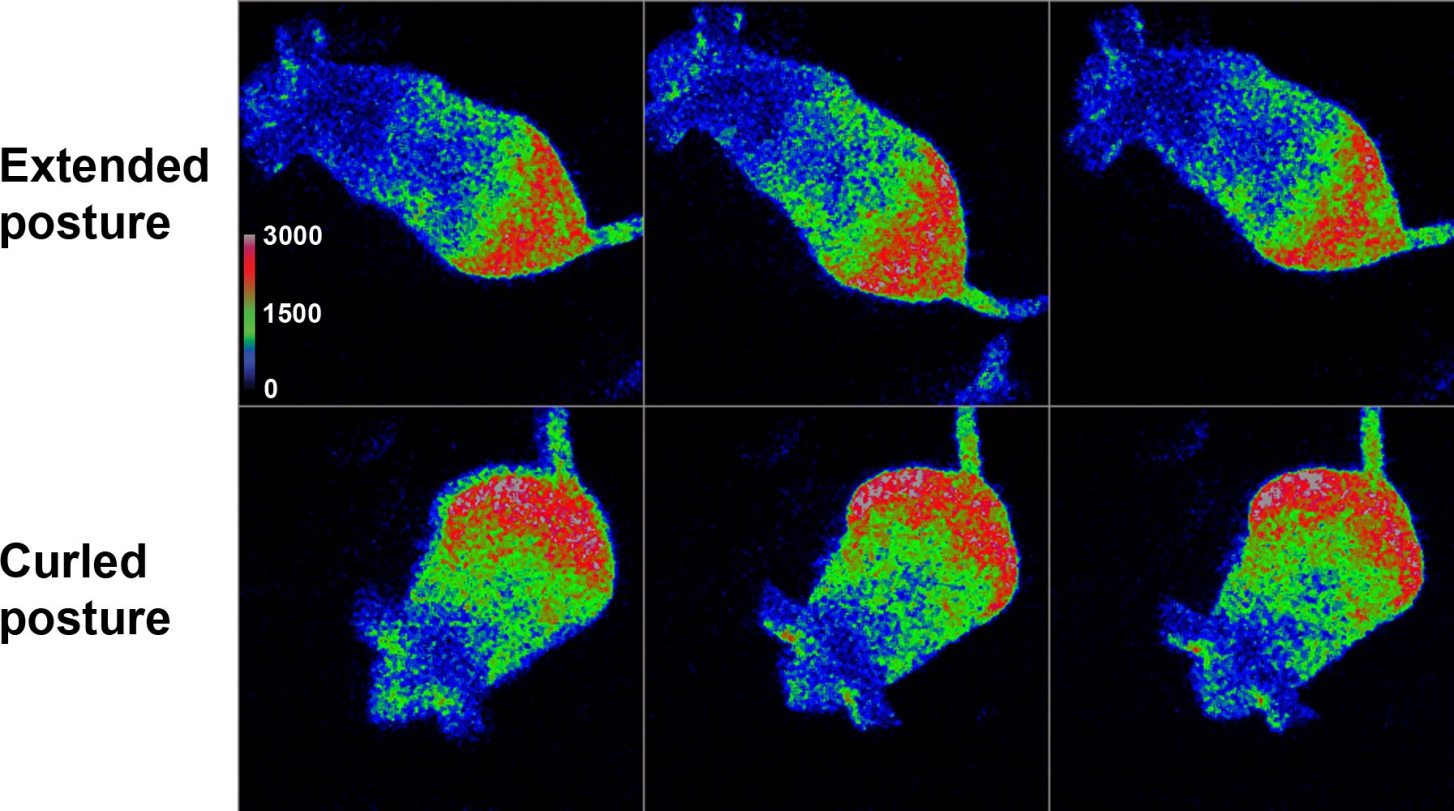
Luciferin assimilated by drinking enables long-term bioluminescence imaging. Shown is a female non-anesthetized, freely moving Hr-Per1 mouse imaged after oral luciferin delivery (1 mM luciferin in apple juice). Frames were captured with 0.5 second exposures within a 15-min imaging sequence and selected by eye according to body shape contours. Top row: in the extended posture. Bottom: curled posture. The intensity range is in ADUs with 20x EM gain.

Luciferin delivered orally through voluntary drinking is an effective way to image mice without inducing stress from handling, injections, or anesthesia [45]. To test whether this method is suitable for long-term imaging that can last for several hours Hr-Per1 mice were moved to a recording chamber and provided luciferin in apple juice (S1 Fig). We tested two similar EMCCD cameras that provide fast and sensitive imaging to determine whether they can be used to minimize image blurring during BLI. Mice in a freely moving state were imaged successfully and anatomical details were in sharp focus after luciferin injected by IP (Fig 1) or after oral luciferin delivered though spontaneous drinking (Fig 2). Specific body shapes and postures could be readily distinguished. Camera exposure time and EM gain were varied to reduce blurring from movement. Not surprisingly, mice could be imaged with longer exposures while they were inactive, particularly with the oral luciferin delivery method that allowed for longer recording sessions to monitor Per1 gene expression. When mice were moving, exposures as short as 0.1 sec were effective for imaging. Because EMCCD cameras lack a mechanical shutter they do not produce noise during image acquisition that might induce a startle response from the mice.

**Fig 3:**
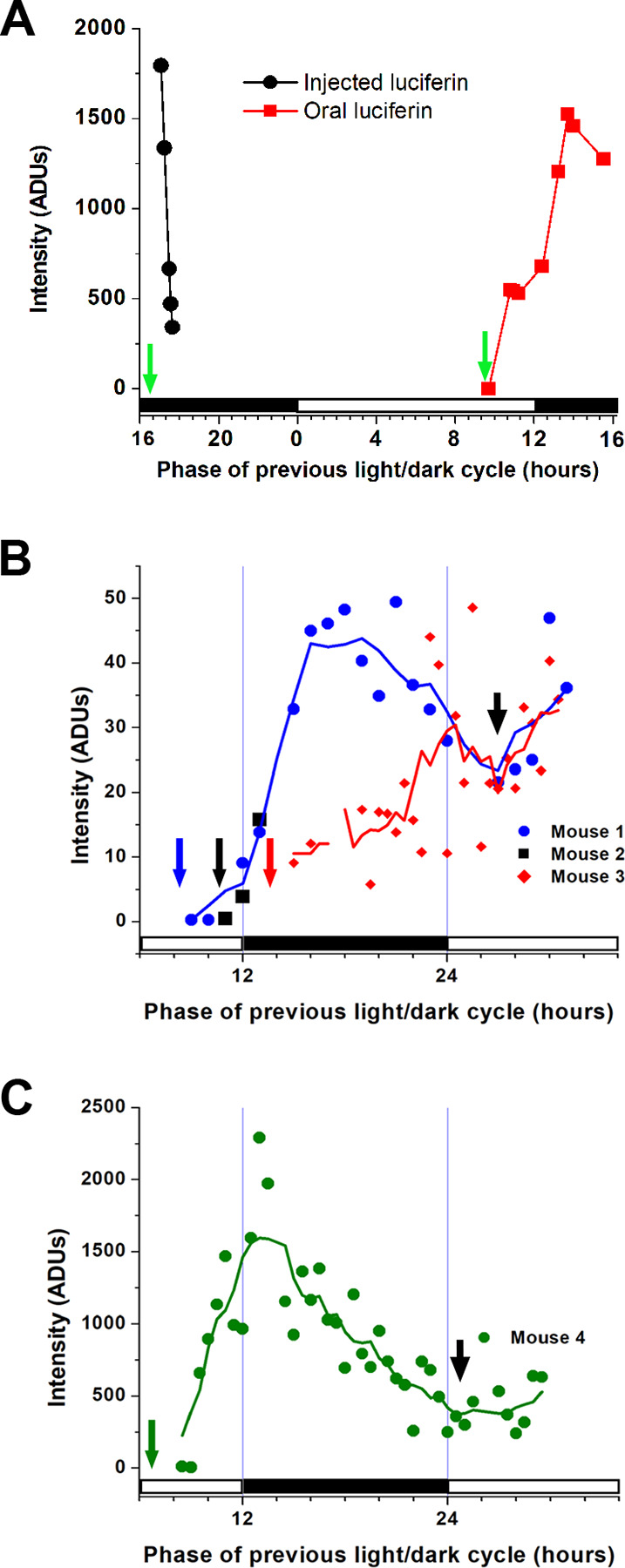
Enhanced bioluminescence signal stability following oral versus injected luciferin delivery. **A:** Average maximum signal from a freely moving female mouse imaged with the Evolve EMCCD camera after luciferin injection (first arrow) shows the predicted rapid decline over the next hour. The first point was adjusted to correct for different camera settings. Oral luciferin provided to the same mouse (second arrow) before the time when circadian activity begins, near projected dusk (hour 12), produced a rapid and sustained signal. Bars below show the light and dark phases of the light cycle before the recording. **B:** Average pixel intensity of three freely moving mice provided with oral luciferin at three time points around the end of the day (long arrows) and imaged with the non-EMCCD camera (CH360) and 60-minute exposures to integrate the signal. Luciferin delivered before projected dusk produced a rapid rise in signal in long (Mouse 1) and short (Mouse 2) recordings. Luciferin provided after projected dusk produced a slower increase in signal (Mouse 3) that converged with the level achieved by Mouse 1 including the dip in signal following projected dawn (short arrow). Shown is integrated whole animal bioluminescence without any selection for the posture of the mouse or its position within the field of view. **C:** A fourth mouse imaged with the non-EMCCD camera and provided with oral luciferin five hours below the projected night shows a similar temporal pattern of bioluminescence including the dip in signal after projected dawn. Lines in **B** and **C** are 5-point running averages.

As expected, image collection following luciferin delivery by injection was practical during only a short time window. As observed here and in other studies [36], injection produced maximal bioluminescence in about 5 minutes, which was suitable for a sharp image, as shown by the male Hr-Per1 mouse in Fig 1B. However, the signal decayed rapidly to half this value within about 25 minutes, as shown in Fig 3A. The luciferase signal reached a peak at 5 and 7 minutes after injection in two additional Hr-Per1 mice (one male and one female), which provided adequate imaging over less than an hour during freely moving imaging. Initially, we qualitatively evaluated images of anesthetized mice and freely moving mice after luciferin injection, according to image intensity and anatomical clarity. Bright tissues and structures near the skin surface that express Per1, such as the submandibular salivary gland (SMG) and testes [51–53], were clearly visible in freely moving and anesthetized mice injected with luciferin (Fig 1B, 1C & 1H). Furthermore, the non-EMCCD camera (CH360) that was used for BLI of anesthetized mice (Fig 1B) was as effective as the EMCCD camera (Evolve) used for imaging freely moving mice (Fig 1C–1H).

Because Per1 is expressed rhythmically as a molecular component of the circadian clock we tested whether the clock’s effects on the bioluminescence signal could be minimized by imaging mice at nearly the same phase of the circadian cycle and 12–13 minutes after luciferin injection. To perform this optimization bioluminescence from anatomical regions of seven anesthetized female Hr-Per1 mice was compared quantitatively. All imaging was completed between ZT (Zeitgeber time) 18:14 and 19:43, where dusk (light offset) occurred at ZT 12:00. The average pixel intensity was measured within eye-drawn regions-of-interest. Significant differences in gene expression could be detected by this approach with the SMG luminescing brighter than the right front paw (p<0.01) and right rear paw (p<0.05) by Tukey HSD post hoc test after one-way ANOVA (F(2, 18) = 10.06, p = 0.00272) with signals of 4597 (±2876 SD), 1610 (±876), and 2762 (±1663) ADUs, respectively.

### Distinct body positions of Hr-Per1 mice imaged after luciferin delivery by drinking

After providing luciferin for drinking, the first signal appeared as early as 10 min, in which case images were captured with 5-sec exposures, 20x gain, and 4 x 4-pixel binning using EMCCD cameras. Subsequent images were brighter and were captured with 0.5 or 0.1 sec exposures, 20x or 400x gain, and 2 x 2 binning (Fig 2). The animal’s entire outline was visible as it moved indicating that, unlike conventional BLI methods using anesthetics, simultaneous real-time monitoring of gene expression and behavior is possible using this freely moving technique. Average integrated signal intensities from the mouse in Fig 2 are also shown in Fig 3A. Imaging sessions were performed at intervals lasting between 15 minutes and 10 hours, after which the drinking tube volume was checked, and the mouse was examined with red light before imaging resumed. The average consumption rate of luciferin in apple juice was about 1 ml/hour, and it also varied across the circadian cycle with greater drinking during the time corresponding to the night of the previous light/dark cycle. We observed no aversion to drinking luciferin in apple juice or water.

Mice were given access to luciferin with apple juice in the drinking tube at several times around subjective dusk, the time of day corresponding with when darkness began in the previous light/dark cycle but projected forward into the condition of constant darkness used during imaging (hour 12 in Fig 3). Per1 gene expression in rodent tissues such as lung, liver, and skeletal muscle is known to increase throughout the daylight portion of the light cycle and then fall during the night [54], which likely contributed to the magnitude of the signal and the momentary drop shortly after projected dawn (Fig 3B and 3C) when Per1 expression typically reaches a daily minimum. For example, as shown in Per1 mRNA expression data of the online CircaDB database [55]. Images with strong signal intensity were also captured from an additional freely moving Hr-Per1 mouse given oral luciferin and imaged briefly. The average maximum pixel intensity (956.7 ±153.9 ADUs) across six time points over 3¼ hours was similar to the recorded signal from the other mice.

### Improved bioluminescence measurements after selecting images according to body posture and location in the chamber

After luciferin was provided for drinking, imaging was performed for at least 20 hours without substantial signal degradation, in contrast to the rapid signal loss following luciferin injection. The maximum BLI duration possible using this method is currently not known, but our results suggest that gene expression may be monitored non-invasively without intervention by imaging for more than one day. The strong signal present at the end of all long-term imaging sessions, using any of the three cameras, suggested that luciferin is adequately maintained by drinking and that imaging could be continued for much longer by replenishing the supply. The chamber design allows access to the luciferin reservoir from outside the chamber (S1 Fig). The long duration of the imaging sessions allowed unperturbed mice to be imaged well after handling or other stress-inducing disturbances introduced near the start of the experiment. The oral luciferin delivery therefore has the potential benefit of providing images well after any effects from initial physiological stress have subsided.

**Fig 4:**
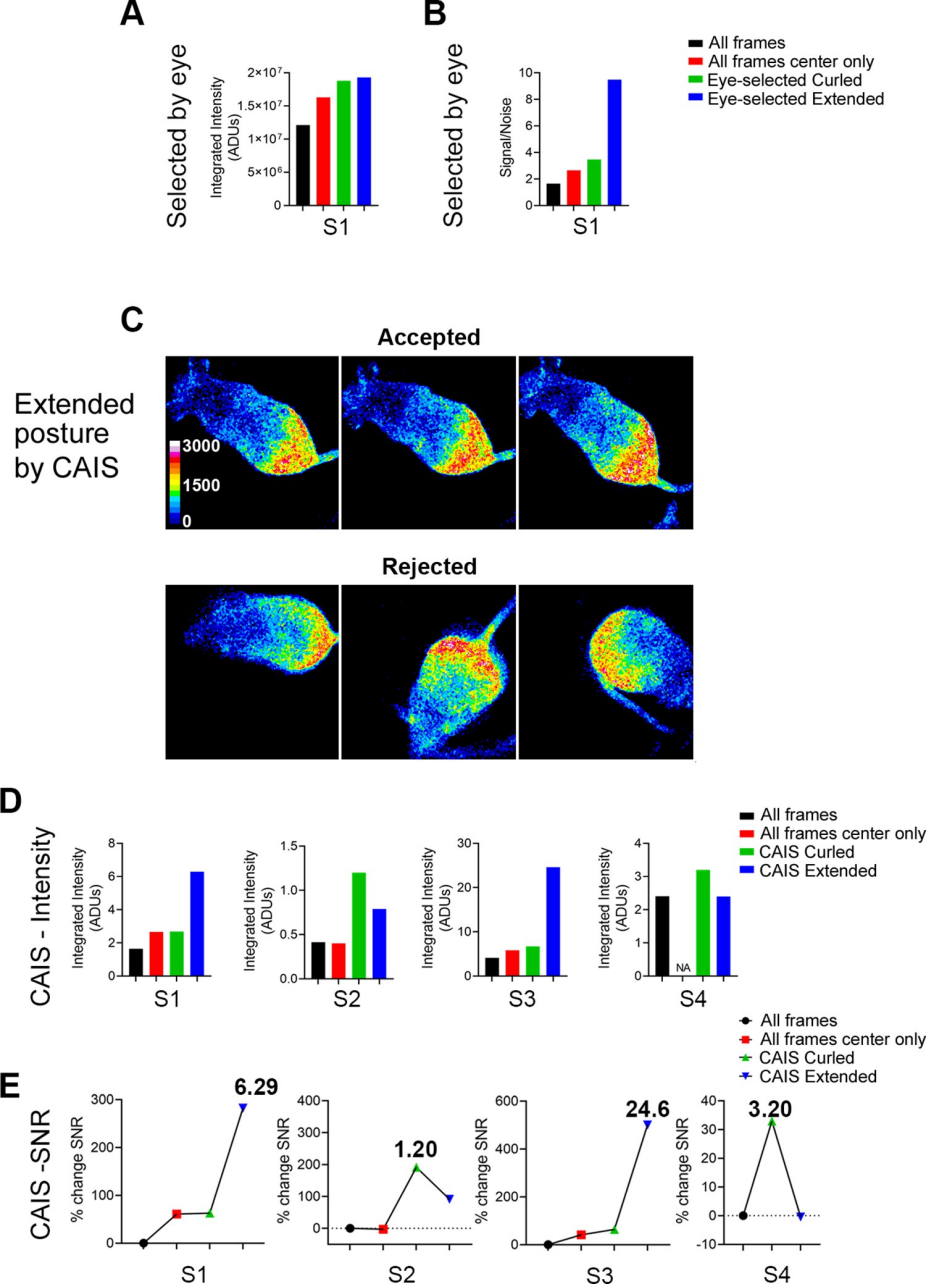
Improved signal-to-noise ratios of bioluminescence in Hr-Per1 mice after selection for body position and posture. A: Average integrated intensity of frames in the entire time series segment S1 (All frames) is shown with three subgroups: after selection for frames with center-of-mass of intensity within the image central quadrant (center only) and in two subgroups of image frames selected by eye according to two body postures (curled and extended). Measured intensity increased in the three derived subgroups. B: Signal-to-noise ratio (SNR) increased several-fold in the subgroups relative to the All frames (full frame) group. C: Images of a freely moving mouse given oral luciferin and selected by computer-aided image selection (CAIS) as meeting criteria for the extended posture (accepted) or not (rejected). Some frames chosen through CAIS were also detected among the eye-selected images, for example top row, right and Fig 2, top row, middle. D: Integrated intensity (ADUs divided by 10^7^) of images from the same time series segment and three more grouped by computer-aided image selection (CAIS) based on average object roundness for the two eye-selected groups. E: SNR increased overall. S1, S2, and S4 were collected from mice given oral luciferin. S3 is the same mouse as in S1 but given injected rather than oral luciferin.

One challenge with imaging freely moving mice we considered is that some of the light signal might be reduced through reflection or absorption by the wall of the chamber, preventing it from reaching the lens. Another obvious concern is that the mice present different body areas to the camera as they change their posture within the recording chamber (Fig 2), reducing the reliability of measurements from both integrated-signal (whole animal) and region-of-interest (specific body area) intensity measurements. Integrated-signal imaging is just a summing of all light captured in the camera exposure (all pixels in the frame) and is essentially luminometry, which does not provide spatial information. To explore how to minimize the variability in the integrated signal we examined the fluctuations in integrated intensity in relation to posture and position relative to the edges of the camera’s field of view. The integrated intensity was examined with images of mice in multiple postures (poses) and was found to vary with posture (Figs 2 and 4C) or when mice were near the wall of the chamber.

To test whether the measured average integrated intensity would be higher when mice were near the center of the chamber, indicating signal loss from proximity to the wall, we sorted through BLI time series collected from three freely moving mice provided luciferin by drinking. One of these was also imaged after luciferin was injected IP rather than by drinking. We then averaged only the frames when the center-of-mass, calculated with ImageJ, was within the central quadrant of the image, 128 x 128 pixels. In two of the mice, imaged with the Evolve camera, this selection for the “centered” mouse images increased the integrated intensity and signal-to-noise ratio (SNR). SNR was calculated from the integrated intensity averaged across multiple frames divided by the standard deviation of the mean integrated intensity across the same frames (Fig 4). Except where noted, all subsequent image calculations were restricted to frames meeting this center-of-mass criterion. The third of these mice (series S4, Fig 4) was imaged with the iXon3 camera and 4 x 4-pixel binning, providing a 128 x 128-pixel image, so selection by center-of-mass was not used.

Additional procedures were evaluated for their ability to increase the average integrated intensity and SNR. First, images from a series of 164 frames collected from a freely moving female mouse were grouped by eye into two subseries based on whether the mouse was in one of two postures that were distinctly different (Fig 2). The two resulting time series had 8 frames of the mouse when extended and 8 frames when curled. The average integrated intensity in the two subgroups increased relative to the original series (Fig 4A), and SNR was improved by up to about 3-fold (Fig 4B).

We examined three additional stacks of images collected from this mouse after oral luciferin delivery, all collected during the projected night (ZT 12–24 of the previous light/dark cycle). These measurements included pixels from the entire frame rather than just the central quadrant. The image stack collection ranged from one to 15 minutes in duration using 0.5 second exposures captured every second. The average image intensities in the all-frames, extended, and curled groups using the eye-selected criteria were 3.96 (±0.593), 4.24 (±0.735), and 4.5 (±1.016) x10^7^ (±SD), respectively (n = 3 stacks). The average SNR for these three image stacks were 7.09 (±0.762), 98.6 (±146). and 25.1 (±12.1), respectively. Again, the average intensity and SNR increased in the extended and curled groups, indicating the benefits from selecting frames of similar pose was not restricted to a particular image stack from this mouse.

### Applying computer-aided image selection

To test whether the semi-automated CAIS approach would provide improvements that were near those achieved using the two eye-selected postures we relied on a series of ImageJ procedures to select for frames with extended and curled postures. The five mice used for CAIS analysis were imaged between ZT 10:17 and 17:34, which is just before or during their projected night, the time when mice are most active. The first time series analyzed was the same entire (All frames) image stack used in Fig 4A. As before, the center-of-mass of the total integrated intensity was used to determine the animal’s position and to restrict the selection to frames with the object center located within the central quadrant. Images were converted to binary using the thresholding feature and then roundness was calculated. Specific details of the image processing and roundness measurements used for the CAIS procedure are provided in S1 File.

Average roundness values for the eye-selected curled and extended groups were 0.597 ±0.086 SD and 0.411 ±0.048, respectively, n = 8 each. The roundness of the 144 usable frames of the entire series was 0.613 ±0.144. Roundness values were then used to create two acceptable ranges, one for extended and one for curled postures, to screen the original time series automatically (Fig 4C). As a proof-of-principle to test CAIS, these two ranges were set arbitrarily to within plus and minus one standard deviation from the curled and extended means, which provided two non-overlapping ranges. When chosen by CAIS, the measured integrated intensity and SNR again increased in the resulting extended and curled subseries (S1 in Fig 4D and 4E). The same criteria for high and low roundness were then used to create curled and extended subseries from images of the same mouse given luciferin by IP injection and two additional mice given oral luciferin (one male and one female). Again, the improvement in SNR was substantial, over 4-fold for one time series (S3 in Fig 4D).

The SNR of the second mouse, imaged with the Evolve camera, was surprisingly low relative to the other mice (S2 in Fig 4E). We attributed that to both a lower average signal and the very long time series analyzed. This additional source of variance contributing to the noise should be removable by restricting the time series to shorter intervals, around one or two minutes, that still provide enough sampling of body position and posture to allow their contributions to noise to be removed by CAIS. The very short exposure times used (0.1–0.5 sec) would be suitable for capturing these many samples. when images from the third mouse given oral luciferin were processed by CAIS, the intensity and SNR of the extended group, but not the curled group, improved relative to the all-frames (center only) group (S4 in Fig 4). This male was imaged with the iXon3 camera.

Two additional freely moving female mice were given oral luciferin and imaged with the iXon3 camera while evaluating use of the photon-counting mode (PCM) option it provides. Although curled and extended poses were observed, these images had a lower signal intensity than expected and were mostly composed of discrete pixels rather than smooth objects probably because this mode misses counts when the object is not a very dim light source [56]. Only single photons are often registered at higher photon flux rates. Consequently, we applied an alternative approach using the ImageJ Gaussian blur function to blend neighboring pixels (S3 Fig), and then ellipses of varying shape were fit to thresholded images of each stack, as described in S1C in S1 File. Roundness, which is the minimum ellipse axis divided by the maximum ellipse axis, was calculated to sort the frames of the image stack into curled and extended groups by applying the same roundness criteria derived from the eye-fit values of stack S1 (0.363–0.459 and 0.511–0.683). The analyzed stacks contained 20 frames for mouse 4 and 120 for mouse 5. The analysis was not restricted to the central quadrant because of the pixel binning of mouse 5. Again, the average integrated intensity and SNR increased in the extended and curled groups relative to the all-frames group (Table 1).

**Table 1:** Alternative CAIS analysis of freely moving mice imaged with photon counting mode.

	Average integrated intensity across frames*			SNR			Pixel binning
Mouse	All Frames	Curled	Extended	All Frames	Curled	Extended	
4	5.48 ±3.91** (n = 18)	4.03 ±2.50 (n = 13)	9.27±4.62 (n = 5)	1.40	1.61 (14.7%)	2.01 (43.2%)	2x2
5	83.4 ±1.14 (n = 120)	85.2 ±0.895 (n = 59)	86.1 ±1.14, (n = 28)	7.32	9.52 (30.1%)	7.55 (3.23%)	8x8

*x10^6^ photon counts

**SD, n is number of usable frames. Percent change in SNR relative to All Frames is included.

The cooled-CCD camera (CH360), which lacks image intensification or electron multiplication at the sensor, was also tested for use in freely moving BLI. Exposure times and on-sensor binning were varied. When the animal was not moving, 10-second exposures were considered optimal for minimizing image blurring while also providing adequate SNR and dynamic range. Because of the low numbers of unblurred images we did not apply CAIS. Typical images captured during the animal’s active phases are shown in S2 Fig, which were acquired 159 min after dusk (lights-off) in the light-dark cycle. Luciferin was supplied in a 35-mm glass petri dish for drinking.

## Discussion

We first confirmed that luciferin delivered through spontaneous drinking could be used effectively to image unanesthetized, freely moving Hr-Per1 mice for several hours. Luciferin was provided through drinking to avoid the stress induced by handling during luciferin injection, anesthesia, or recovery after receiving an implanted pump to deliver luciferin [17, 19]. This simple yet elegant approach provided long-term imaging of luciferase bioluminescence throughout the Hr-Per1 mice, as was reported recently through freely moving BLI or other mice [35]. Furthermore, the proposed method of restricting imaging in darkness mostly to the animal’s night is useful in preventing stress-evoked effects on gene expression, including induction of Per1, a key component of the molecular circadian clock [57–59]. Although this study was not intended to measure circadian rhythms in Per1 expression, its rhythmic expression patterns in mouse tissues have been described previously. Instead, it was to reduce noise in BLI and develop an overnight procedure for sampling part of the circadian cycle so that circadian influences on imaging longer physiological and behavioral processes can be minimized.

A circadian rhythm in Per1 gene expression has been reported previously in freely moving mice when bioluminescence was recorded from the brain through an optical fiber [60]. Similarly, the organs of rodents typically have high Per1 expression during the night that declines towards dawn [54, 61]. Circadian rhythms in Per1 expression were previously measured in freely moving mice using a sophisticated 2-camera 3-dimensional BLI system [42]. Interestingly, rhythms measured in that study agreed with the current results, showing bioluminescence minima in the skin and other structures just after the time when dawn would have occurred if the mice had remained in the prior light cycle, rather than being in continuous darkness. These minima near subjective dawn are also visible in the rhythmic Per1 mRNA expression data from adrenal gland, kidney, liver, lung, and skeletal muscle of mice available at the CircaDB website: http://circadb.hogeneschlab.org/ [55]. We therefore conclude that the circadian clock modulated the signal recorded here through control of the Per1 gene. It might also result from alterations in luciferin intake though a rhythm in drinking as described below. Furthermore, imaging was limited to only one circadian cycle rather than at least two that would be needed to confirm the presence of a circadian rhythm and confirm its period.

The orally administered luciferin provided a more sustained signal than luciferin injection because the body’s luciferin levels were replenished through additional drinking. Mice readily drink luciferin delivered in water [45], and the cost of oral luciferin delivery is not excessive based on the approximate 20 ml fluid consumption per mouse each day. Although apple juice encouraged mice to drink quickly, appealing flavored water could also be effective. Previously, we found that mice show luciferase expression within about one hour of introduction to luciferin in water or apple juice and that they can be imaged repeatedly over several months using this method with no deleterious effect [45]. Furthermore, we previously reported that the distribution pattern of bioluminescence among body regions was similar following injected and oral luciferin delivery, although injected luciferin produced a higher but transient signal.

Animals clearly have circadian rhythms in drinking behavior [62] suggesting a possible effect on luciferin uptake. The proposed imaging procedure made use of the tendency of the mice to drink the luciferin during the night. Circadian urine production [63] suggested that luciferin clearance might affect signal strength following injected or oral luciferin. Nevertheless, rhythms in clearance were not apparent in one study in which luciferin was delivered by osmotic pumps [19]. In a separate study, plasma luciferin concentrations were reported to be stable over 24 hours when it was delivered by an external pump [42]. Therefore, rhythmic loss of luciferin may not be an important confounding variable in BLI. Mice continued to show a minimum in bioluminescence signal corresponding with the reported minimum in the Per1 gene expression rhythm when oral luciferin was provided over a range of hours around dusk even though the maximum signal attained did vary (Fig 3B and 3C).

For imaging experiments concerned only with day-to-day changes, such as tumor growth, the effects on signal strength from circadian rhythms in gene expression, drinking, or possibly excretion can be minimized by scheduling imaging for the same time of day after mice are removed from the light/dark cycle. For the Per1 gene monitored here, oral luciferin delivery first provided near subjective dusk followed by continuous imaging and measurements at the dip following subjective dawn appears optimal for providing the most consistent results while also minimizing imaging time. Initializing a BLI session just before dusk may also be favorable for reducing disruption of sleep while also avoiding a shift in the phase of the circadian clock system from retinal light exposure after dusk when light has the largest effects on rodent circadian pacemakers. Mice in the proposed procedure used here receive the light needed by nocturnal rodents to produce the phase delay of their circadian pacemakers to remain entrained.

We acknowledge that mice will spend more time exploring when placed in a novel environment, and the lack of light at a particular time of day might produce a phase shift as a dark-pulse effect [64]. Nevertheless, this approach seems far better than handling mice and placing them in a novel environment during the middle of the day, which would disturb their sleep and induce locomotor activity and stress hormones at a time when they are normally low. Furthermore, we suggest allowing mice a couple days of entrainment to the light/dark cycle in the home cage between BLI sessions to maintain synchrony within the circadian system. We have not validated this method for monitoring circadian rhythms for more than about a day in darkness, although there is no obvious reason why freely moving BLI combined with oral luciferin delivery and CAIS would fail to be effective for imaging lasting several days.

The near-dawn bioluminescence minimum seems to be the optimal time to make repeatable measurements when Per1 expression is imaged by any freely moving method. Because various treatments used in BLI experiments could shift the circadian clock or alter its amplitude it is advisable to initially measure signals throughout the cycle because the waveform of the rhythm could be altered. If BLI is performed using expression of a different transgene or reporter gene whose possible circadian clock control has not been previously described, then at least 48 hours of imaging should be performed initially to determine when the clock may be influencing the signal.

It is very likely that additional factors also increased the variability of the captured signal, in particular differences in mouse position and posture that would alter the light captured as different areas of brightness were presented to the camera. Therefore, two approaches were tested to reduce variability and increase signal strength—fast, sensitive cameras and rapid collection of images that could be automatically evaluated and sorted by CAIS to provide more accurate BLI. The results indicated that SNR can be increased substantially after corrections are made for posture and size through eye-selected sampling or CAIS. Also, average signal intensity often increased in the subseries groups based on posture when images that were not fully capturing the body signal were excluded by CAIS. Even this simple procedure for size, shape, and position discrimination used here was able to produce substantial improvements in SNR, indicating finer gene expression changes should be discernable. Although the photon counting mode did not seem appropriate for imaging these mice, because of their brightness, it was useful for confirming that a simpler, alternative CAIS routine can also discriminate between poses and improve intensity measurements and SNR.

More advanced posture-recognition software than the basic ImageJ program used here would be expected to exceed these improvements by identifying more body positions for tracking and discrimination to further increase accuracy and acuity. One possibility is a recently introduced image analysis program for automated scoring of mouse interactions [65]. Another recent technology allows BLI of brain activity in freely moving mice engaged in social interactions [66].

Although both EMCCD cameras provided adequate signal intensity with almost no blurring, a cooled non-EMCCD camera designed for longer exposures, minutes to hours, was also effective for imaging freely moving mice. Although suitable image capture was restricted to intermittent resting states, images devoid of blurring were attainable with less sophisticated technology than EMCCD cameras, making this method accessible to a broad range of users. The cooled-CCD camera was, however, most effective when used as a luminometer to monitor whole body bioluminescence by summing pixel intensities in each frame, which was sufficient for revealing the known circadian rhythm in Per1 expression. In most cases, this camera provided images with indistinct mouse edges, because of movement, that were not suitable for the roundness measurements needed for the CAIS procedure. It is likely that infrared imaging with a second camera could be used in conjunction with the cooled-CCD camera to independently detect static mouse poses for use in CAIS of BLI images.

Unlike the total light emission recordings during whole-body luminometry, BLI provides additional information about differential gene expression throughout the body. For example, the ratio of signals from two or more points can indicate differences in gene expression in tissues, such as the elevated SMG versus paw expression shown here in anesthetized Hr-Per1 mice. A similarly high SMG signal was reported when BLI was used to examine live transgenic mice expressing a fusion protein of the circadian clock gene Per2 and luciferase under control by the Per2 promoter [17]. During BLI, the skin’s light-scattering properties can obscure precise localization of internal light sources [67]. In addition, some of the signal from Hr-Per1 mice likely originated from the skin, which is known to express Per1 [61]. The testes also produced a bright signal that may be useful as a control for BLI studies of clock-controlled genes because, unlike most organs, this gland lacks a circadian clock [51–53]. They might also be useful for evaluating luciferin availability in mice independent of clock timing effects. The testes signal could have caused differences in whole-body signal measurements between male or female mice, but we did not detect a noticeable effect of sex with the small sample sizes used here.

Obviously, the ability to selectively quantify different body location signals can be more meaningful than integrated intensity measurements that lack spatial information, for example when evaluating tumor growth or organ-specific gene responses to a drug treatment. Furthermore, whole-body BLI can provide spatial information about animal behavior not obtainable from intensity measurements derived by imaging luciferase that is only expressed at a specific body site. Nevertheless, the integrated bioluminescence signal from freely moving mice can be recorded from the entire body even when the signal originates from a single body location, as shown previously when a luciferase reporter gene was expressed through viral infection [68].

Because light emitted from firefly luciferase and luciferin reacting within tissues is near 600 nm [69] we likely experienced less light scattering than imaging methods based on shorter wavelength luciferase or bacterial lux bioluminescence, which has maximal emission near 490 nm [70]. Further refinements in imaging are occurring based on luciferin analogs and selectively mutated firefly luciferase emitting in the near-infrared that enable imaging of deep internal structures [69, 71]. One method is reported to be as much as a thousand-times brighter than firefly luciferase when a modified luciferin with higher catabolic activity is also used, which allows measurements of individual cells in mice [71]. Finally, the lack of toxicity of various luciferin analogs during BLI should be tested and compared with the far greater knowledge derived from firefly luciferin used in live cells.

## Conclusions

New technologies for monitoring gene expression in freely moving mice will benefit from luciferin delivery to mice through drinking while selected images of matching postures are collected and collated automatically for analysis. Gene expression can be measured in conscious mice engaged in normal behaviors without stressors from anesthesia or surgery and with minimal handling and isolation that can alter behavior, the reporter gene activity producing the signal, and study outcomes.

We found that a simple pattern-matching computer algorithm can readily sort images into stacks of acceptable frames according to body posture and position. These images can be collected from static mice or ones in motion using either standard cooled-CCD cameras or high-speed EMCDD cameras, respectively. Images can be collected sequentially over several hours allowing large numbers of images to be analyzed retrospectively or immediately to provide higher accuracy. The CAIS approach could be applied to other types of imaging based on, for example, fluorescence, near infrared, or thermal imaging in which the contours of the animal are visible. The CAIS method is essentially a trainable protocol, relying on initial estimates of the two ranges of roundness provided by the user. Additional refinements in image discrimination are possible, and more user interaction could be added to tune the selection criteria for specific transgenic mouse bioluminescence patterns, animal shape, etc., ultimately relying on artificial intelligence for selection and data curation.

The starting time for luciferin access by the mice was varied over several hours, and a consistent pattern in expression emerged suggesting that, with this transgenic mouse, intensity measurements may be most consistent when obtained after projected dawn several hours after mice were left undisturbed to engage in normal behaviors throughout the night. The known circadian oscillation in the Per1 transgene used in this study suggested when influences on gene expression measurements from the noise produced by circadian clocks can be minimized. Essentially, daily imaging sessions at the same circadian phase could provide more consistent results in BLI studies of gene expression when it is influenced directly or indirectly by the circadian clock. When this method is used to monitor expression of a gene controlled by the clock, we advise determining whether any experimental treatments also alter the circadian system. Combining these timing adjustments for clock effects with CAIS and oral luciferin delivery produces a simple and reliable method for monitoring gene expression in freely moving mice exposed to minimal stressors.

## Supporting information

S1 Fig Imaging chamber design. A glass optical window rests on four 19 mm-long urethane foam spacers attached to the rim of the cylinder. A hole provides additional airflow and is also explored by the mouse as a nose-poke hole. A standard stainless steel sipping tube is attached and connected with a stopper to a 15-ml plastic centrifuge tube containing water or apple juice with luciferin. Ground corn cob bedding and mouse chow pellets are provided.
(TIF)

S2 Fig Bioluminescence images of freely moving mouse captured with a cooled-CCD camera. **A:** Brightfield view of one Hr-Per1 mouse captured with red LED light and a 50-msec exposure. The sipper tube is shown at left. Also shown are food pellets and the bedding. **B & C:** Representative images of a freely moving Hr-Per1 mouse acquired without use of an image intensifier or on-chip electron multiplication. Images were taken with 10-sec exposures approximately an hour after providing oral luciferin in the 35-mm dish, visible with the mice. The tail and torso could be captured without blurring when the animal was at rest. Images were selected within a 1-min sequence. Binning was used at the sensor (4 x 4 pixels) to maximize light signal and reduce the read-noise component of images. Intensity shown as pseudocolor are in analog-to-digital units (ADUs) of the camera after bias subtraction.
(TIF)

S3 Fig Applying alternative CAIS to freely moving mice imaged with photon counting mode. **A**: One frame from the image stack used for freely moving mouse 4 after the despeckling procedure. **B:** The same image after applying a Gaussian blur with 9-pixel radius and a best-fit ellipse to determine roundness. The intensity range is between 200 and 300 photon counts and is displayed by 16 colors.
(TIF)

S1 File Steps used to select objects in freely moving mouse images. (DOCX)
