# Supplementary figures and images for: Using bioluminescence to image gene expression and spontaneous behavior in freely moving mice
Source: PLoS One. 2023 Jan 20;18(1):e0279875. doi: 10.1371/journal.pone.0279875 (PMC9858005; doi:10.1371/journal.pone.0279875)

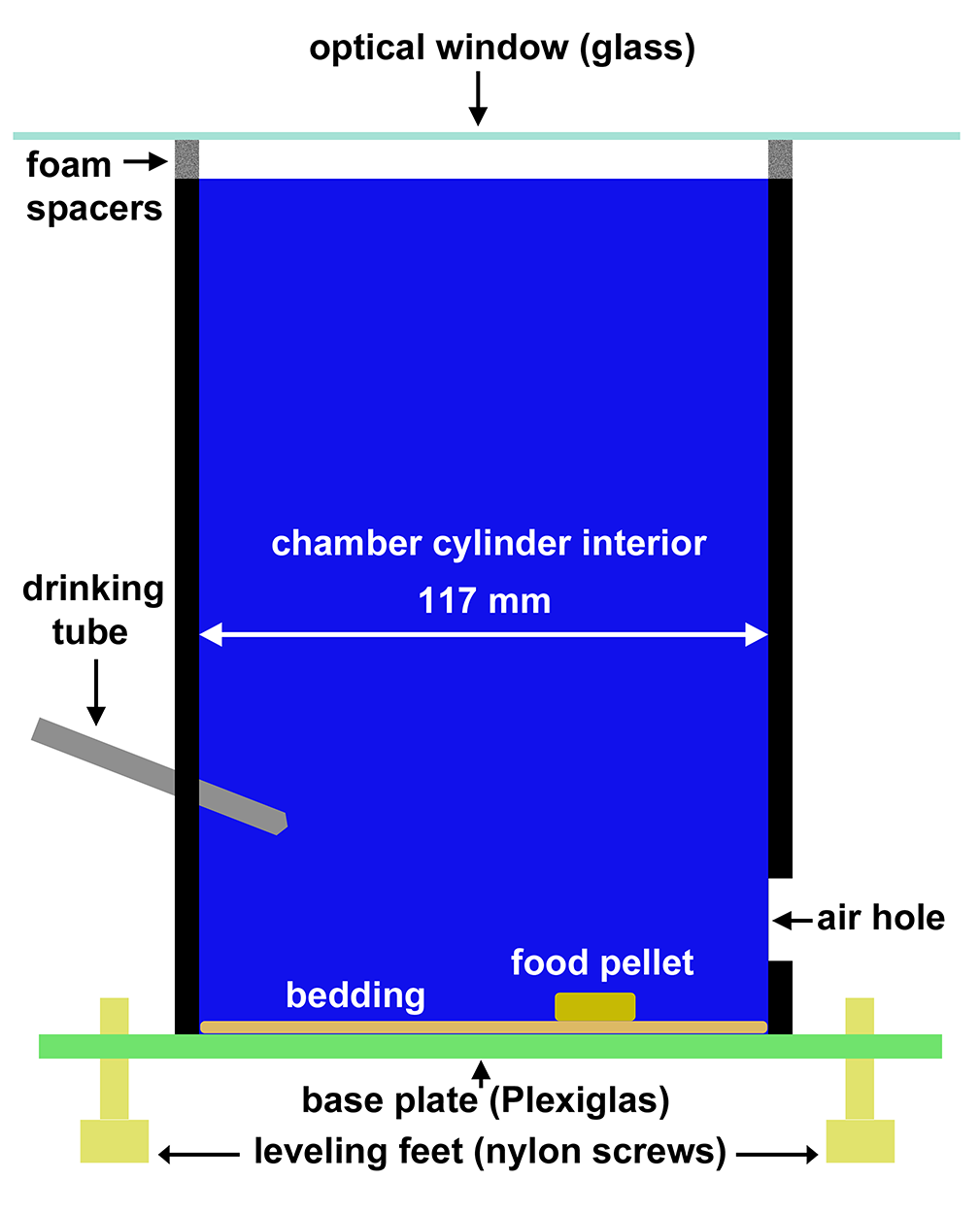

Supplement: S1 Fig — A glass optical window rests on four 19 mm-long urethane foam spacers attached to the rim of the cylinder. A hole provides additional airflow and is also explored by the mouse as a nose-poke hole. A standard stainless steel sipping tube is attached and connected with a stopper to a 15-ml plastic centrifuge tube containing water or apple juice with luciferin. Ground corn cob bedding and mouse chow pellets are provided. (TIF) [file pone.0279875.s001.tif]

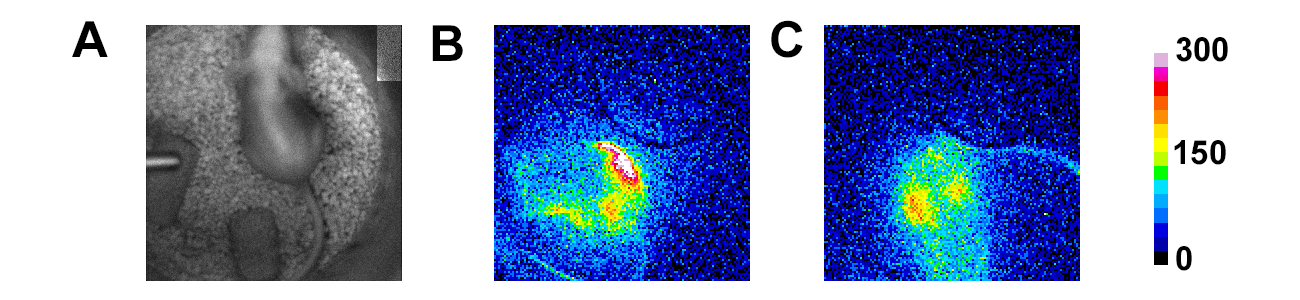

Supplement: S2 Fig — A: Brightfield view of one Hr-Per1 mouse captured with red LED light and a 50-msec exposure. The sipper tube is shown at left. Also shown are food pellets and the bedding. B & C: Representative images of a freely moving Hr-Per1 mouse acquired without use of an image intensifier or on-chip electron multiplication. Images were taken with 10-sec exposures approximately an hour after providing oral luciferin in the 35-mm dish, visible with the mice. The tail and torso could be captured without blurring when the animal was at rest. Images were selected within a 1-min sequence. Binning was used at the sensor (4 x 4 pixels) to maximize light signal and reduce the read-noise component of images. Intensity shown as pseudocolor are in analog-to-digital units (ADUs) of the camera after bias subtraction. (TIF) [file pone.0279875.s002.tif]

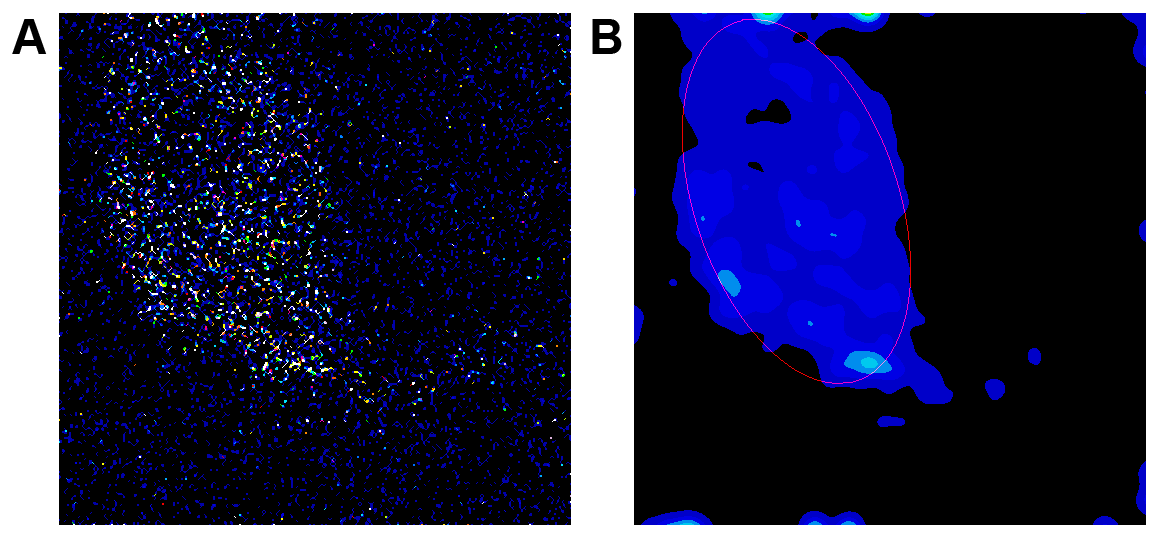

Supplement: S3 Fig — A: One frame from the image stack used for freely moving mouse 4 after the despeckling procedure. B: The same image after applying a Gaussian blur with 9-pixel radius and a best-fit ellipse to determine roundness. The intensity range is between 200 and 300 photon counts and is displayed by 16 colors. (TIF) [file pone.0279875.s003.tif]
