# Supplementary material for: Using bioluminescence to image gene expression and spontaneous behavior in freely moving mice
Source: PLoS One. 2023 Jan 20;18(1):e0279875. doi: 10.1371/journal.pone.0279875 (PMC9858005; doi:10.1371/journal.pone.0279875)
Supplement: S1 File — (DOCX) [file pone.0279875.s004.docx]

**Supplemental 1. Steps used to select objects in freely moving mouse images.**

Frames were analyzed by position, roundness, and integrated intensity using these steps in ImageJ:

**Equations**
Initially processed raw image stack = Median filter of (raw image stack - average bias image)

S/N = Average Integrated Intensity/standard deviation of Average Integrated Intensity.

**Flow chart of image analysis procedures based on posture**

A) Process first raw image stack

↓

B) Select postures by eye and sort into Curled and Extended stacks.

↓ ↓

Curled stack Extended stack

↓ thresholding ↓ thresholding

Binary Curled stack Binary Extended stack

↓ ↓

Analyze Particles Analyze Particles

↓ ↓

Average Roundness Average Roundness

↓ ↓

C) Create stack subsets from Initially Processed Stack based on frame numbers in Curled and Extended stacks.

Curled stack (grayscale) Extended stack (grayscale)

↓ ↓

Integrated Intensity & SD Integrated Intensity & SD

↓ ↓

S/N S/N

D) Select subsets from Curled and Extended stacks based on Center of Mass.

Curled stack subset Extended stack subset

↓ thresholding ↓ thresholding

Binary Curled stack Binary Extended stack

↓ ↓

Analyze Particles Analyze Particles

↓ ↓

Average Roundness Average Roundness

↓ ↓

E) Create stack subsets from Initially Processed Stack based on frame numbers in Curled and Extended stacks.

Curled stack (grayscale) Extended stack (grayscale)

↓ ↓

Integrated Intensity & SD Integrated Intensity & SD

↓ ↓

S/N S/N

**CAIS**

F) Initially process a new image stack

↓ thresholding

Convert to Binary stack

↓

Analyze Particles

↓

G) Find Roundness of Initially Processed Stack.

↓

H) Sort images in Initially Processed Stack into Curled or Extended stacks based on whether their Roundness falls within a range extending one SD above and below the average eye-selected Curled and Extended Roundness values (B above).

↓

I) Create stack subsets from Initially Processed Stack based on frame numbers in Curled and Extended stacks.

Curled stack subset (grayscale) Extended stack subset (grayscale)

↓ ↓

Integrated Intensity Integrated Intensity

↓ ↓

S/N S/N

J) Select subsets from Curled and Extended stacks based on Center of Mass.

Curled stack subset Extended stack subset

↓ thresholding ↓ thresholding

Binary Curled stack Binary Extended stack

↓ ↓

Analyze Particles Analyze Particles

↓ ↓

Average Roundness Average Roundness

↓ ↓

K) Create Substacks from Initially Processed Stack based on frame numbers in Curled and Extended stacks.

Curled stack (grayscale) Extended stack (grayscale)

↓ ↓

Integrated Intensity Integrated Intensity

↓ ↓

S/N S/N

**Details of analysis procedures**

A. For Integrated Density (intensity) measurements:

1. Create a stack from the sequence of individual images in the time series (Image>Stacks>Images to Stack).

2. Subtract the camera bias, for example in this case 508, from all images in the stack (Process>Math>Subtract). Alternatively, subtract from the image stack the average image of a stack of bias images (Process>Image Calculator).

3. Use Median Filter (Process>Filters>Median) with 2-pixel radius to remove spurious charge and to allow more accurate thresholding.

4. Use Measure Stack (Plugins>Stacks>Measure Stack) after setting these parameters in Analyze>Set Measurements: area, min & max gray value, center of mass, integrated density, mean gray value, modal gray value, centroid, and stack position.

5. Save image stack and Results as a text file.

B. Additional steps for measuring roundness:

6. Create a binary image (Process>Binary>Make Binary) by using Threshold with the lowest minimum that includes the head and torso in the object and within as many frames as possible. The highest value, maximum pixel intensity, is found by moving the slider to encompass the object of interest in one frame in red. Set the selected values and deselect Calculate for each image.

7. Use Analyze>Analyze Particles with minimum particle (object) size as 2000 pixels. Select Including Holes and Show Outlines. Save image stack and Results. Check for errors such as part of the head region missing or more than one particle (object) counted in a frame, and then repeat with a different minimum particle size if necessary.

8. Save Results and image stack.

Equations and descriptions for these procedures are in the ImageJ help file.

Only the Plugins in the standard installation were used.

C. Alternative steps for measuring roundness when image resolution is low.

1. Use Process>Noise>Despeckle to remove some background counts.

2. Use Process>Filters>Gaussian Blur with sigma (radius) of 9 to 16 to expand pixels.

3. Threshold the image stack (Image>Adjust>Threshold) Use default threshold, for example 210-232, and select Calculate threshold for each image.

4. Use Analyze>Analyze Particles with an appropriate minimum particle (object) size, for example 5000-infinity pixels. Select Circularity of 0-1, Show Ellipses, Display results, Clear results, Summarize, Include holes.

Check for errors such as more than one particle (object) counted in a frame, multiple ellipses, and then repeat these procedures with a larger minimum particle size if necessary.

5. Use Min and Max axes values of the ellipse in the Summary of the results to calculate roundness for all frames in the stack. (Roundness=Min/Max).

6. Use Measure Stack to calculate the Integrated Density (intensity) for the original stack after bias and background removal.

7. Use the roundness values to sort the frames into Extended or Curled subset stacks. Then, use the Integrated Density of the frames represented in these stacks to calculate the mean intensity and the SD of the mean.

8. Calculate SNR as mean/SD for the all-frames, extended, and curled groups.
